# Formative Evaluation of Consumer-Grade Activity Monitors Worn by Older Adults: Test-Retest Reliability and Criterion Validity of Step Counts

**DOI:** 10.2196/16537

**Published:** 2020-08-18

**Authors:** Stephanie A Maganja, David C Clarke, Scott A Lear, Dawn C Mackey

**Affiliations:** 1 Department of Biomedical Physiology and Kinesiology Simon Fraser University Burnaby, BC Canada; 2 Faculty of Health Sciences Simon Fraser University Burnaby, BC Canada; 3 Division of Cardiology Providence Health Care Vancouver, BC Canada; 4 Centre for Hip Health and Mobility University of British Columbia Vancouver, BC Canada

**Keywords:** aged, gait, mobility limitation, exercise, movement, wearable electronic devices, mobile phone, reproducibility of results, bias, dimensional measurement accuracy

## Abstract

**Background:**

To assess whether commercial-grade activity monitors are appropriate for measuring step counts in older adults, it is essential to evaluate their measurement properties in this population.

**Objective:**

This study aimed to evaluate test-retest reliability and criterion validity of step counting in older adults with self-reported intact and limited mobility from 6 commercial-grade activity monitors: Fitbit Charge, Fitbit One, Garmin vívofit 2, Jawbone UP2, Misfit Shine, and New-Lifestyles NL-1000.

**Methods:**

For test-retest reliability, participants completed two 100-step overground walks at a usual pace while wearing all monitors. We tested the effects of the activity monitor and mobility status on the absolute difference in step count error (%) and computed the standard error of measurement (SEM) between repeat trials. To assess criterion validity, participants completed two 400-meter overground walks at a usual pace while wearing all monitors. The first walk was continuous; the second walk incorporated interruptions to mimic the conditions of daily walking. Criterion step counts were from the researcher tally count. We estimated the effects of the activity monitor, mobility status, and walk interruptions on step count error (%). We also generated Bland-Altman plots and conducted equivalence tests.

**Results:**

A total of 36 individuals participated (n=20 intact mobility and n=16 limited mobility; 19/36, 53% female) with a mean age of 71.4 (SD 4.7) years and BMI of 29.4 (SD 5.9) kg/m^2^. Considering test-retest reliability, there was an effect of the activity monitor (*P*<.001). The Fitbit One (1.0%, 95% CI 0.6% to 1.3%), the New-Lifestyles NL-1000 (2.6%, 95% CI 1.3% to 3.9%), and the Garmin vívofit 2 (6.0%, 95 CI 3.2% to 8.8%) had the smallest mean absolute differences in step count errors. The SEM values ranged from 1.0% (Fitbit One) to 23.5% (Jawbone UP2). Regarding criterion validity, all monitors undercounted the steps. Step count error was affected by the activity monitor (*P*<.001) and walk interruptions (*P*=.02). Three monitors had small mean step count errors: Misfit Shine (−1.3%, 95% CI −19.5% to 16.8%), Fitbit One (−2.1%, 95% CI −6.1% to 2.0%), and New-Lifestyles NL-1000 (−4.3%, 95 CI −18.9% to 10.3%). Mean step count error was larger during interrupted walking than continuous walking (−5.5% vs −3.6%; *P*=.02). Bland-Altman plots illustrated nonsystematic bias and small limits of agreement for Fitbit One and Jawbone UP2. Mean step count error lay within an equivalence bound of ±5% for Fitbit One (*P*<.001) and Misfit Shine (*P*=.001).

**Conclusions:**

Test-retest reliability and criterion validity of step counting varied across 6 consumer-grade activity monitors worn by older adults with self-reported intact and limited mobility. Walk interruptions increased the step count error for all monitors, whereas mobility status did not affect the step count error. The hip-worn Fitbit One was the only monitor with high test-retest reliability and criterion validity.

## Introduction

### Background and Rationale

In Canada, almost 90% of older adults (aged ≥65 years) do not meet the national physical activity recommendation of ≥150 min per week of moderate-to-vigorous aerobic physical activity [1]. Worldwide, physical inactivity is linked to an increased risk of type 2 diabetes, cardiovascular disease, colon cancer, osteoporosis, and postmenopausal breast cancer [2-7]. In addition, physically inactive older adults are at risk for falls, dependence in activities of daily living, and mobility limitation [8]. Mobility limitation affects approximately 30% of older adults in Canada and the United States and is linked to adverse health outcomes, including mobility disability and nursing home admission [9-11]. Older adults with a mobility limitation could especially benefit from physical activity interventions and corresponding physical activity monitoring [9-11].

Monitoring physical activity in older adult populations in both research and clinical settings is useful for several reasons: to detect longitudinal changes in physical activity levels [12], to determine the effects of interventions [13-19], to assess adherence to physical activity programs [14,18], to quantify daily physical activity patterns [20,21], and to motivate older adults to meet physical activity goals [22]. Consumer-grade activity monitors are a relatively affordable type of wearable technology that count steps in addition to quantifying other metrics of physical activity behavior. Older adults accept activity monitors, find them helpful for motivation, and often prefer them over simple pedometers [23].

To use a commercial-grade activity monitor to count the steps of older adults in research and clinical settings, the measured step counts must be reliable and valid [24]. If step counts exhibit poor test-retest reliability (eg, measurement errors vary from day to day), this limits the ability to detect changes in an individual’s physical activity over time [25]. If step counts exhibit poor criterion validity (eg, systematic under or over counting of steps), this may lead to incorrect conclusions about the effectiveness of physical activity interventions or the effects of physical activity on health outcomes [24].

### Prior Work

Substantial evidence indicates that step counts from consumer-grade activity monitors exhibit high interdevice reliability and criterion validity in healthy adults [26]. However, age-related changes in gait may affect the precision and accuracy of step counting [27]. To this end, emerging evidence from studies of older adults shows that the criterion validity of step counts from consumer-grade activity monitors is high during short-distance walks conducted in controlled laboratory settings at walking speeds >0.8 m per second [28]. However, consumer-grade activity monitors tend to overcount the steps of older adults during longer distance walking in free-living conditions [28-31] and undercount the steps when older adults walk with an assistive device, such as a walker [8,28,32,33].

Important gaps in evidence remain to be addressed. First, the test-retest reliability of step counts from consumer-grade activity monitors has not been evaluated in older adults [28]. Second, the influence of self-reported mobility limitation on the reliability and validity of activity monitor step counts in older adults has not been investigated. Finally, although aspects of the walking environment, including interruptions to continuous walking, have been suggested to influence the reliability and validity of step counting in adults [34-37], the effects of interruptions on walking have not been studied in older adults.

### Study Aims

This study was motivated by our need to select a consumer-grade activity monitor for a randomized trial of a physical activity intervention for older adults, and the necessary data on the reliability and validity of step counts were not available. Thus, the purpose of this study was to evaluate the reliability and validity of step counts from consumer-grade activity monitors when worn by community-dwelling older adults during overground walking. The first aim was to determine how the *test-retest reliability* of step counting varied across 6 consumer-grade activity monitors and was affected by the presence of self-reported mobility limitations. The second aim was to determine how the *criterion validity* of step counting varied across 6 consumer-grade activity monitors and was affected by the presence of self-reported mobility limitations and walk interruptions.

## Methods

### Recruitment

Older adults were recruited through a variety of methods: study flyers posted around the community (eg, libraries, community and seniors’ centers, and coffee shops); presentations by researchers and fitness instructors to groups of older adults (eg, at exercise classes); advertisements in local newspapers and recreation program guides; and email messages to previous research participants, fitness class attendees, and university alumni.

Individuals were eligible for inclusion, determined through telephone screening, if they were aged 65 years or older, community dwelling, and able to speak, read, and write English. We purposely recruited individuals with and without self-reported limited mobility. Individuals were classified as having limited mobility if they self-reported difficulty walking one-quarter mile (2 to 3 blocks) outside on level ground or going up a flight of stairs (about 10 steps) without resting [10,38,39]; otherwise, they were classified as having intact mobility. Individuals were excluded if they reported an inability to walk 400 meters independently or scored below 26 (indicative of cognitive impairment) on the Montreal Cognitive Assessment [40,41]. If the Physical Activity Readiness Questionnaire for Everyone [42] indicated any medical contraindication to physical activity, the individual had to receive physician approval to participate in the study.

The study was approved by Simon Fraser University’s Research Ethics Board and the University of British Columbia’s Clinical Research Ethics Board. All participants provided verbal consent to telephone screening and written informed consent to participate in the study.

### Descriptive Measures

Participant demographics including age, sex, racial background, level of education, and smoking history were obtained through a self-report questionnaire. Participants also self-rated their health compared with others of a similar age on a 5-point scale (excellent, good, fair, poor, or very poor). Height was measured with a portable stadiometer (seca GmbH & Co. model 217 1821009), and weight was measured with a digital scale (seca GmbH & Co. model 874 1321009). The BMI (kg/m^2^) was then calculated. Lower extremity physical function was assessed using the Short Physical Performance Battery (SPPB) [10,11], which involved tests of standing balance, 6-meter gait speed, and chair stands to assess leg strength. The SPPB was scored out of 12, with a higher score indicating better function. Additional descriptive information was collected through self-report questionnaires, including physical activity, comorbidities (Functional Comorbidity Index) [43], and computer and cellphone use.

### Outcome Measures

#### Activity Monitors

Six activity monitors were evaluated (Table 1). Three monitors were worn on the hip: Fitbit One, Misfit Shine, and New-Lifestyles NL-1000 Pedometer. The other 3 monitors, the Fitbit Charge, Garmin vívofit 2, and the Jawbone UP2, were worn on the wrist. The settings for each monitor were customized to the participant’s height, weight, and age and were simultaneously placed on the nondominant side of their body according to the manufacturer’s instructions. Wrist-worn monitors were randomized to their location on the arm (closest to the wrist, middle, or farthest from the wrist). Two of the hip-worn monitors were randomly assigned to 1 of 2 sites, either closer to the belly button or to the hip. The position of the New-Lifestyles NL-1000 hip-worn monitor was not randomized and was always placed halfway between the belly button and the hip, according to the manufacturer’s recommendation. The randomization procedure was performed before testing.

**Table 1:** Description of activity monitors.

Monitors	Manufacturers	Body placement	Digital display	Step counting instruments
Fitbit Charge	Fitbit, San Francisco, California, United States	Wrist	Yes	Three-axis accelerometer
Fitbit One	Fitbit, San Francisco, California, United States	Hip	Yes	Three-axis accelerometer
Garmin vívofit 2	Garmin, Olathe, Kansas, United States	Wrist	Yes	Three-axis accelerometer
Jawbone UP2	JAWBONE, San Francisco, California, United States	Wrist	No	Three-axis accelerometer
Misfit Shine	Misfit, Burlingame, California, United States	Hip	No	Three-axis accelerometer and magnetometer
New-Lifestyles NL-1000	New-Lifestyles, Lee’s Summit, Missouri, United States	Hip	Yes	Piezoelectric pedometer

#### Walking Trials

**Figure 1:**
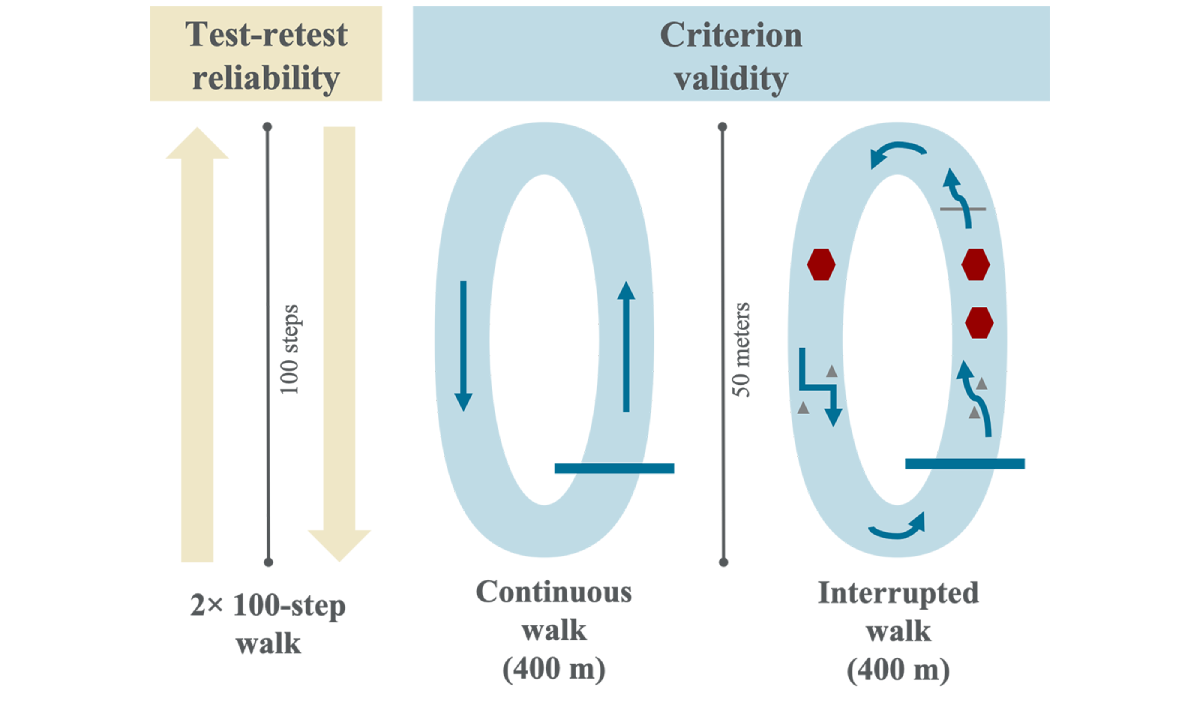
Walking trials completed in a level hallway. For criterion validity testing, participants walked 4 laps of the continuous and interrupted courses to reach 400 meters.

Participants completed 4 walking trials in a long hallway (Figure 1). Testing was conducted on weekends to avoid weekday foot traffic, and signs were displayed to minimize disruptions. For each walk, one researcher instructed the participant to start and stop walking, whereas another researcher timed the walk with a stopwatch and recorded the time to complete the walk. During the walks, the 2 researchers walked slightly behind the participants and counted their steps using tally counters. Tally counts were used as the criterion measure, which is common in activity monitor assessment [24,37,44]. When discrepancies occurred between steps counted by the 2 researchers, the median value was used and rounded up to the nearest whole number unless one researcher believed they miscounted the steps, in which case the other researcher’s number was used. In total, 6 activity monitor step counts were recorded immediately before and after each walk.

For all trials, participants were instructed to walk at their preferred walking speed, defined as a comfortable speed that they could maintain for the duration of the walk. To prevent fatigue, participants were provided with adequate rest time between the walks (5 to 15 min). The 4 walking trials were typically completed within 1 hour, within which approximately 15 min of walking was completed (approximately 1 min for each 100-step walk and approximately 5 to 7 min for each 400-meter walk).

The first 2 reliability walks (RW1 and RW2) required the participant to walk 100 steps (Figure 1). A researcher notified the participants when they had 5 steps left to walk and provided a verbal countdown to the end of the walk. If a participant did not walk exactly 100 steps on their first walk, the participant was instructed to walk the same number of steps for their second walk.

For the 400-meter continuous walk (CW), a 100-meter course was defined using pylons (Figure 1). Participants completed 4 laps of the course without stopping, beginning, and ending at the same point on the course.

Research suggests that the walking environment and interruptions can affect the validity and reliability of step counts from activity monitors [34-36], so a 400-meter interrupted walk (IW) was included to mimic the conditions of daily walking more closely than the CW (Figure 1). Participants walked the same 400 meters as in the CW, but 7 interruptions were incorporated into each lap using additional pylons and signs. These interruptions included an S-curve, 2 consecutive 5-second stops, object avoidance (stepping over a tree branch), a sharp turn to change the direction, one 5-second stop, 2 successive 90-degree angle turns, and an additional sharp turn. In completing 4 laps, participants encountered each interruption 4 times for 36 interruptions in total.

### Measurements

Walking trial step counts for each activity monitor were calculated by subtracting the step count recorded at the beginning of the walk from the step count recorded at the end of the walk (eg, end of CW step count − beginning of CW step count = CW step count). To account for participants walking a different number of steps per trial, all step counts were converted to step count percent errors, which were calculated as follows:

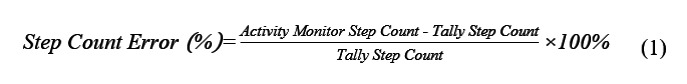



Step count percent errors closer to zero are more desirable. Positive step count percent errors indicated that the activity monitor was overcounting steps relative to the tally count (criterion), whereas negative step count percent errors indicated undercounting relative to the tally count.

### Statistical Analysis

#### Sample Size

We calculated that a total sample size of 34 participants (17 within each group of intact mobility and limited mobility) would provide 80% statistical power to detect an effect size of 5% step count error within each group with significance level alpha of .05, assuming that the SD in step count error was similar to what was observed in our pilot data (SD 3.3, n=5 young adults). We aimed to recruit 20 participants within each group to account for potential missing data.

#### Descriptive Analysis

Descriptive data for participant characteristics are presented as means and SDs for normally distributed continuous variables and medians and IQRs for skewed continuous variables. Judgments of normality were based on the visual inspection of frequency distributions. Categorical variables are reported as frequencies and percentages. To assess differences in descriptive characteristics between the groups with intact and limited mobility, 3 types of statistical tests were used depending on how the data were distributed: independent sample *t* tests for normally distributed continuous variables, Wilcoxon rank-sum test for skewed continuous variables, and chi-square tests for categorical variables. These tests were performed using JMP software (SAS Institute; version 13.1; 2016). Descriptive data for step count errors are presented as means and 95% CI. Statistical modeling was conducted using RStudio version 1.0.136. The family-wise significance level for statistical tests was set at an alpha of .05.

#### Test-Retest Reliability

As a measure of trial-to-trial consistency, the absolute difference between step count percent errors from RW1 and RW2 was calculated as follows:

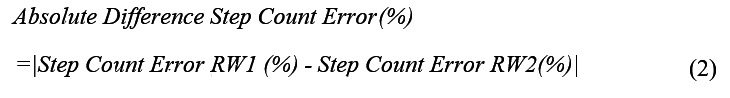



A two-way analysis of variance (ANOVA) was used to assess the effects of the *activity monitor* and *mobility status* on the mean absolute difference in step count percent error between RW1 and RW2. A post hoc analysis of pairwise differences was conducted using the Tukey honest significant difference (HSD) test, where appropriate, which held the experiment-wise error rate constant at an alpha level of .05. To assess the normality of the step count percent error distributions, we visually inspected the quantiles of the distribution, histograms, and density plots and ran a Shapiro-Wilk normality test. Owing to suggestions of nonnormality, we also ran a nonparametric test, Kruskal-Wallis, which produced the same results and led to the same conclusions as the ANOVA. For ease of interpretation, we reported only the results of the ANOVA.

In addition, the standard error of measurement (SEM) was calculated as a descriptive measure of test-retest reliability. SEM was calculated as the SD of the differences between the step count percent errors of RW1 and RW2, divided by the square root of the number of walks, in accordance with Hopkins [25].

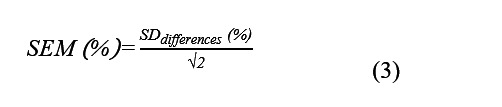



#### Criterion Validity

A three-way ANOVA was used to determine whether the *activity monitor*, *interruptions to walking*, and *mobility status* had effects on the mean step count percent error. A post hoc analysis of pairwise comparisons was conducted using the Tukey HSD test, where appropriate.

Bland-Altman plots [45,46] were produced to assess for systematic bias and limits of agreement in step counts for each activity monitor and for the CW and IW. The mean step count from the 2 measures was plotted on the x-axis (eg, [activity monitor step count]+[tally counter step count]/2), and the error between the 2 measures was plotted on the y-axis (eg, [activity monitor step count]−[tally counter step count]). Reference lines indicate the mean step count error, trend, and 95% limits of agreement (mean +1.96 SD and −1.96 SD).

In accordance with previous studies [33], equivalence testing was conducted to evaluate whether mean step count percent errors were equivalent to a zero step count percent error for each activity monitor and for both 400-meter walks. We defined the equivalence bound as −5.0% to +5.0% step count error, which we deemed to be clinically relevant. Two one-sided *t* tests were conducted to evaluate both sides of the equivalence interval. If there was sufficient evidence to reject both the null hypothesis of the upper threshold (mean error ≤5%) and the null hypothesis of the lower threshold (mean ≥−5%), then the mean step count error was interpreted as *practically* equivalent to a zero step count error.

## Results

### Participants

A total of 36 individuals participated in the study, including 20 with self-reported intact mobility (7 females) and 16 with self-reported limited mobility (12 females; Table 2). The mean age of the participants was 71.4 years (SD 4.7), and the mean BMI was 29.4 kg/m^2^ (SD 5.9). For most characteristics, there were no significant differences between the groups with intact and limited mobility. However, the group with limited mobility had significantly slower gait speed than the group with intact mobility for the 6-meter (*P*<.001) and continuous 400-meter (*P*<.001) walks. In addition, the group with limited mobility had a greater number of comorbidities (*P*=.02).

**Table 2:** Participant characteristics.

Characteristics	Groups			*P* value^a^
	Overall (n=36)	Intact mobility (n=20)	Limited mobility (n=16)	
Female, n (%)	19 (53)	7 (35)	12 (75)	.02
Age (years), mean (SD)	71.4 (4.7)	73.1 (3.7)	71.6 (5.8)	.40
Weight (kg), mean (SD)	82.0 (16.8)	78.1 (17.4)	87.0 (15.1)	.11
BMI (kg/m^2^), mean (SD)	29.4 (5.9)	27.7 (4.4)	31.5 (7.0)	.07
White, n (%)	25 (69)	13 (65)	12 (75)	.46
University education, n (%)	16 (44)	9 (45)	7 (44)	>.99
Montreal Cognitive Assessment (of 30), median (IQR)	27 (26-28)	27 (26-27)	28 (27-29)	.03^b^
Smoked previously, n (%)	17 (47)	8 (40)	9 (56)	.33^c^
Good or excellent self-rated health, n (%)	29 (81)	18 (90)	11 (69)	.20
Short Physical Performance Battery (of 12), median (IQR)	11.0 (10.0-11.0)	11.0 (10.0-12.0)	10.0 (9.8-11.0)	.06^b^
6-meter gait speed (m/s), mean (SD)	1.2 (0.2)	1.3 (0.2)	1.1 (0.2)	<.001
400-meter gait speed (m/s), mean (SD)	1.3 (0.2)	1.4 (0.1)	1.2 (0.2)	<.001
400-meter continuous walk step count, mean (SD)	600 (70)	562 (29)	648 (76)	<.001
400-meter interrupted walk step count, mean (SD)	656 (73)	617 (41)	703 (77)	<.001
Self-reported moderate-to-vigorous physical activity^d^ (min/week), mean (SD)	343 (368)	343 (272)	343 (471)	.10
Self-reported walking (min/week), mean (SD)	208 (197)	240 (208)	167 (182)	.27
Number of comorbidities, median (IQR)	2.0 (0.0-3.0)	0.0 (0.0-2.0)	2.5 (1.0-4.0)	.02^b^
≥1 comorbidity, n (%)	19 (53)	8 (40)	11 (69)	.04
≥2 comorbidities, n (%)	12 (33)	4 (20)	8 (50)	.09^c^
Arthritis, n (%)	13 (36)	4 (20)	9 (56)	.04
Obesity, n (%)	10 (28)	3 (15)	7 (44)	.07
Visual impairments^e^, n (%)	10 (28)	7 (35)	3 (19)	.46
Degenerative disc disease^f^, n (%)	6 (17)	2 (10)	4 (25)	.37
Depression, n (%)	4 (11)	1 (5)	3 (19)	.30
Diabetes (type 1 or 2), n (%)	4 (11)	1 (5)	3 (19)	.30
Osteoporosis, n (%)	3 (8)	0 (0)	3 (19)	.08
Access to a computer with internet, n (%)	31 (86)	17 (85)	14 (88)	>.99
Access to cellphone or smartphone, n (%)	33 (92)	18 (90)	14 (88)	.57

^a^*P* values comparing intact mobility versus limited mobility, from a chi-square Fisher exact test for categorical variables and from an independent sample *t* test for continuous variables.

^b^From a Wilcoxon rank-sum test.

^c^From a chi-square Pearson test.

^d^Moderate-to-vigorous physical activity includes self-reported walking.

^e^For example, cataracts, glaucoma, and macular degeneration.

^f^For example, back disease, spinal stenosis, or severe chronic back pain.

### Test-Retest Reliability

**Figure 2:**
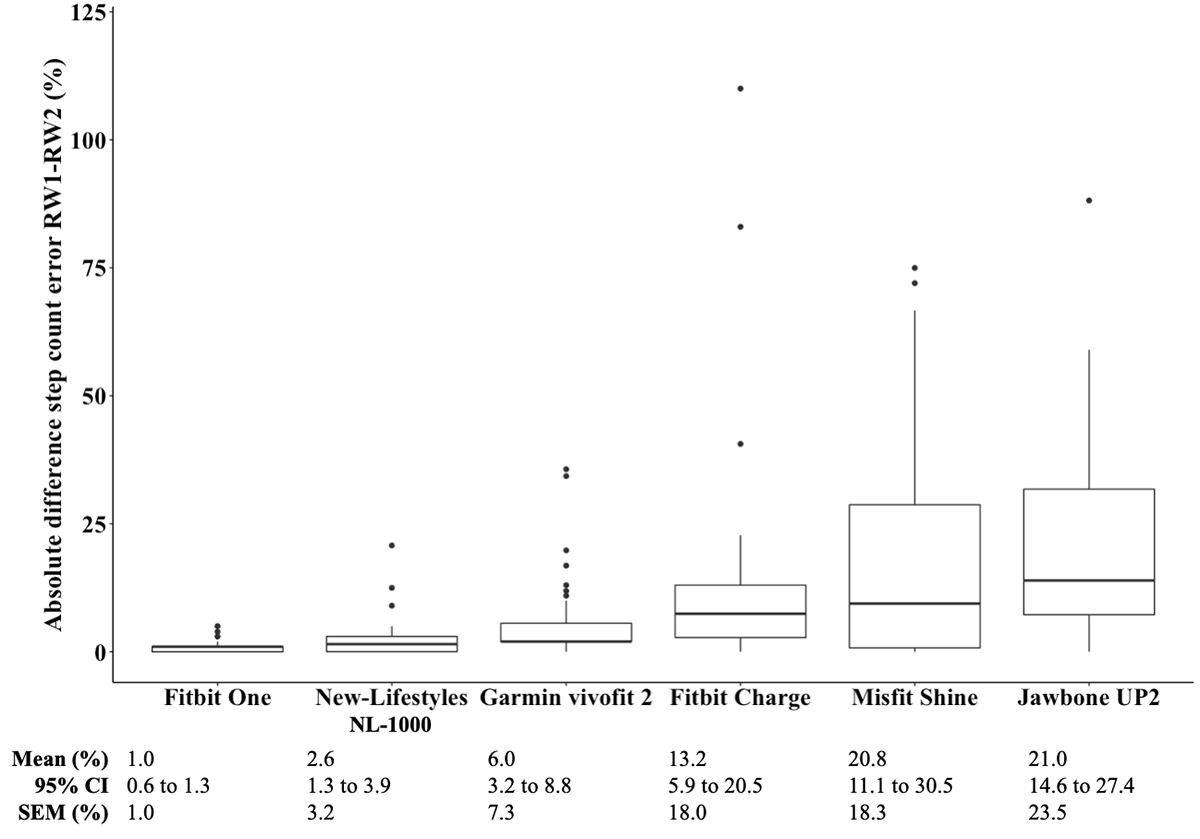
Box plots illustrating median absolute difference in step count percent errors between the 100-step test-retest reliability walk 1 (RW1) and walk 2 (RW2) for the 6 activity monitors (n=36 for all monitors except Misfit Shine, n=28). Central rectangle spans the IQR, and the whiskers represent the inner fence (upper: Q3+1.5×IQR and lower: Q1−1.5×IQR). Fitbit Charge different than Fitbit One (*P*=.02); Misfit Shine different from Fitbit One (*P*<.001), New-Lifestyles NL-1000 (*P*<.001), and Garmin vívofit 2 (*P*=.004); Jawbone UP2 different from Fitbit One (*P*<.001), New-Lifestyles NL-1000 (*P*<.001), and Garmin vívofit 2 (*P*=.002). SEM: standard error of measurement.

We found a significant main effect of the *activity monitor* on the absolute difference between the step count percent errors of RW1 and RW2 (*P*<.001), but we found no main effect of *mobility status* (*P*=.31) and no interaction between the *activity monitor* and *mobility status* (*P*=.29). We found the smallest mean absolute differences in step count percent errors for the Fitbit One (1.0%, 95% CI 0.6% to 1.3%), New-Lifestyles NL-1000 (2.6%, 95% CI 1.3% to 3.9%), and Garmin vívofit 2 (6.0%, 95% CI: 3.2% to 8.8%; Figure 2). Post hoc tests revealed that the Fitbit Charge (*P*=.02), Jawbone UP2 (*P*<.001), and Misfit Shine (*P*<.001) exhibited significantly higher mean absolute differences than the Fitbit One. In addition, the Jawbone UP2 (*P*<.001) and Misfit Shine (*P*<.001) had greater mean absolute differences than the New-Lifestyles NL-1000. Finally, the Jawbone UP2 (*P*=.002) and Misfit Shine (*P*=.004) had greater mean absolute differences than the Garmin vívofit 2. The SEM values ranged from 1.0% (Fitbit One) to 23.5% (Jawbone UP2; Figure 2).

### Criterion Validity

**Figure 3:**
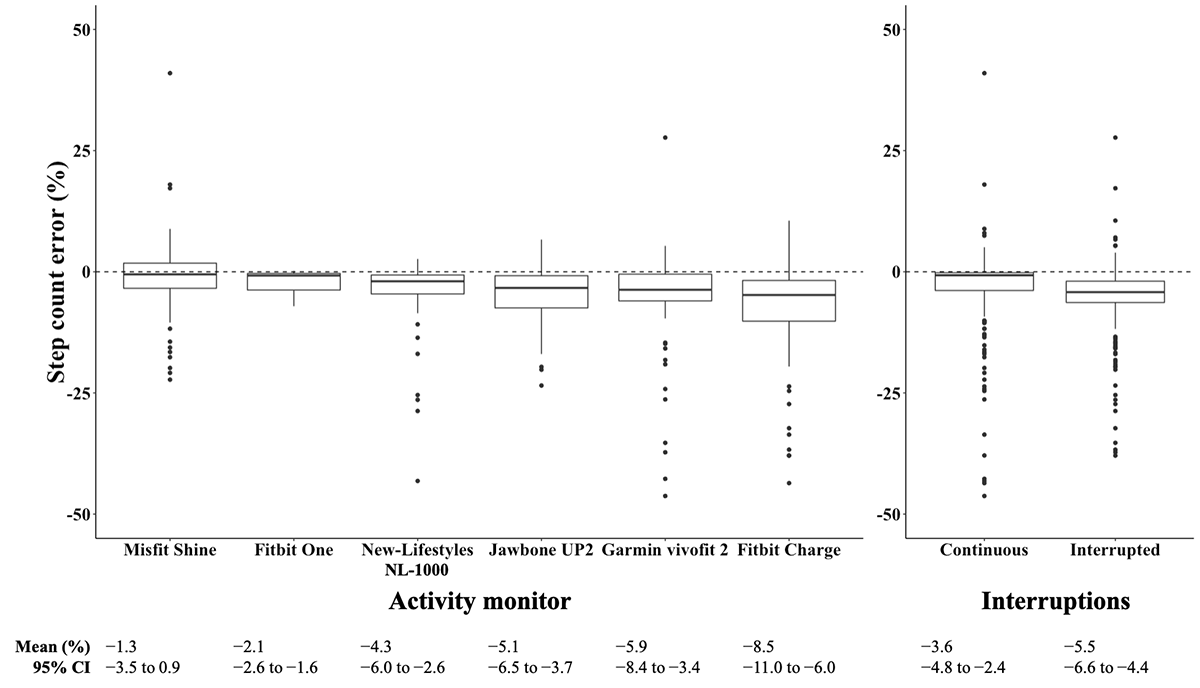
Box plots illustrating median step count percent errors for the 6 activity monitors (n=72 for all monitors except the Misfit Shine, n=67) and for the 2 different 400-meter walks (n=213 for continuous and n=214 interrupted) from 36 older adults. Central rectangle spans the IQR, and the whiskers represent the inner fence (upper: Q3+1.5×IQR and lower: Q1−1.5×IQR). Horizontal dotted lines represent zero step count percent error. Garmin vívofit 2 different from Misfit Shine (*P*=.02); Fitbit Charge different from Misfit Shine (*P*<.001), Fitbit One (*P*<.001), and New-Lifestyles NL-1000 (*P*=.03); interrupted different from continuous (*P*=.02).

The mean (SD) step count from the criterion tally counter on the 400-meter CW was 600 (SD 79) steps and on the 400-meter IW was 656 (SD 73) steps (Table 2). All monitors undercounted steps relative to the criterion (tally) counts (Figure 3), with the Misfit Shine exhibiting the lowest mean step count percent error (−1.3%). We found significant main effects of the *activity monitor* (*P*<.001) and *walk interruptions* (*P*=.02) on step count percent error, but no main effect of *mobility status* (*P*=.65). We observed no interactions between any of the factors. Regarding the main effect of the activity monitor, post hoc tests revealed that the Fitbit Charge (*P*<.001) and Garmin vívofit 2 (*P*=.02) exhibited significantly higher mean step count percent errors than the Misfit Shine. In addition, the Fitbit Charge exhibited a greater mean step count percent error than the Fitbit One (*P*<.001) and the New-Lifestyles NL-1000 (*P*=.03). Regarding the main effect of *interruptions*, the IW resulted in a greater mean step count percent error than the CW (mean difference 1.9%; *P*=.02).

**Figure 4:**
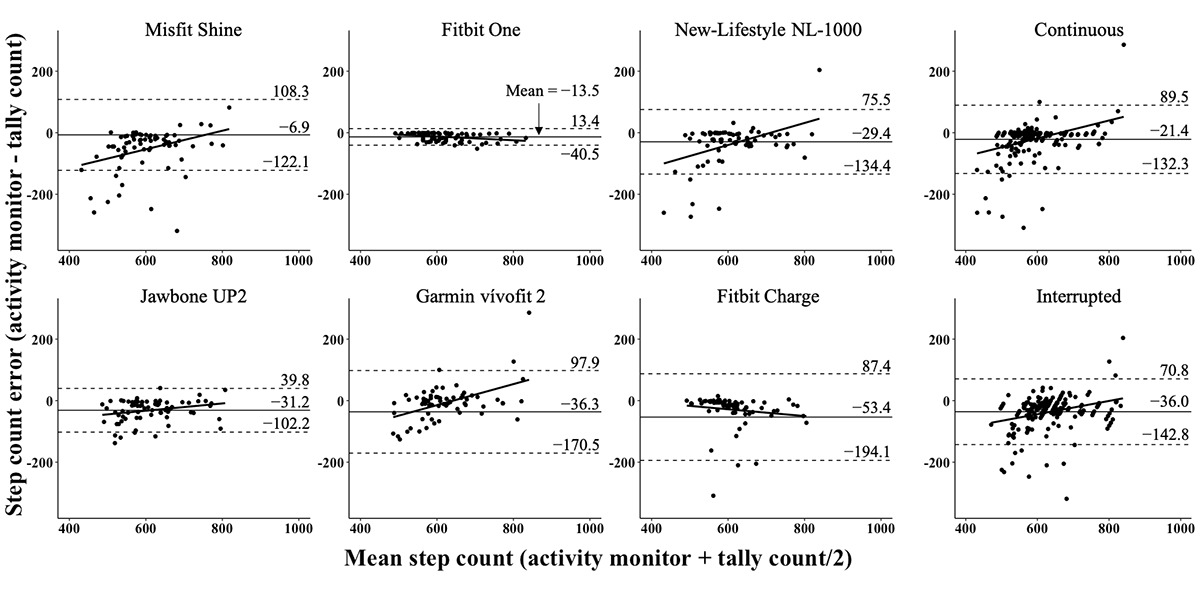
Bland-Altman plots for each activity monitor (n=72 for all monitors except the Misfit Shine, n=67) and for walk interruptions (n=213 for continuous and n=214 for interrupted) compared with the criterion tally counts from 36 older adults. The solid lines represent the mean step count error (horizontal) and line of best fit (trend line). Dotted lines represent the limits of agreement (mean±1.96 SD).

Bland-Altman plots revealed nonsystematic bias across the range of observed step counts for the Fitbit One and Jawbone UP2 (Figure 4). Systematic bias and wide limits of agreement were observed for Misfit Shine, New-Lifestyles NL-1000, Garmin vívofit 2, and Fitbit Charge. In addition, Bland-Altman plots indicated systematic bias across the range of observed step counts and wide limits of agreement for both the CW and IW.

**Figure 5:**
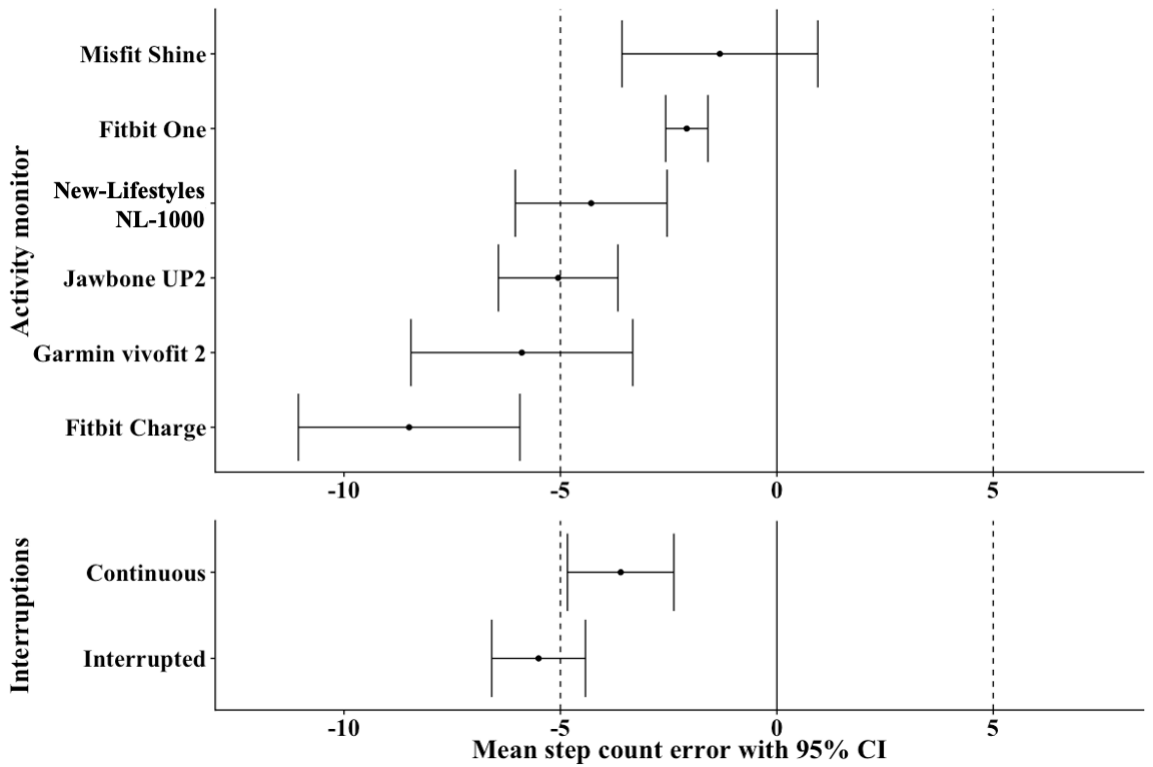
Equivalence testing plots for activity monitor (n=72 for all monitors except the Misfit Shine, n=67) and walk interruptions (n=213 for continuous and n=214 for interrupted). Mean step count errors (%) with 95% CI. Area between dotted vertical lines represents equivalence bounds (+/− 5.0%).

Equivalence tests indicated that the mean step count percent errors of 2 monitors lay within the −5% and +5% equivalence bound, the Fitbit One (*P*<.001) and Misfit Shine (*P*=.001); thus, step counts from these monitors were deemed equivalent to a zero step count percent error (Figure 5). The CW mean step count percent error was statistically equivalent to zero (*P*=.002), whereas the IW mean step count percent error lay outside the equivalence bounds (*P*=.28).

## Discussion

### Principal Findings

Our study aimed to determine (1) how test-retest reliability of step counting by 6 consumer-grade activity monitors was affected by the presence of self-reported mobility limitations in community-dwelling older adults during overground walking and (2) how the criterion validity of step counting by these 6 activity monitors was affected by the presence of self-reported mobility limitations and walk interruptions in community-dwelling older adults during overground walking. We found that test-retest reliability varied across activity monitors (highest for the Fitbit One and lowest for the Jawbone UP2) but was unaffected by the self-reported mobility status. The monitors featured varying degrees of criterion validity, with the Fitbit One exhibiting the highest and the Fitbit Charge, the lowest. Criterion validities were negatively impacted by walk interruptions but were unaffected by self-reported mobility status. The hip-worn Fitbit One was the only monitor that exhibited both high test-retest reliability and criterion validity.

### Comparison With Prior Work

To the best of our knowledge, our study is the first to report on the test-retest reliability of consumer-grade activity monitors in a community-dwelling older adult population [28]. We found that test-retest reliability of step counting, measured by mean absolute percent difference in step count error between repeated 100-step walks, varied across activity monitors. Specifically, only 2 monitors had small mean absolute percent differences in the step count error of less than 5.0%: the Fitbit One and the New-Lifestyles NL-1000. Three monitors (Fitbit Charge, Jawbone UP2, and Misfit Shine) were significantly less reliable than either or both the Fitbit One and New-Lifestyles NL-1000. Finally, the SEM of the Fitbit One was small, within −2.5% and +2.5%, which translates into a between-trial difference of −4.9% and +4.9% step count error in 95 of 100 instances (95% likely range of −4.9% to 4.9%). All other monitors had SEM values indicative of poor reproducibility. Therefore, we conclude that only the Fitbit One had sufficiently high test-retest reliability.

We found that the criterion validity of step counting was affected by both the activity monitor and walk interruptions during 400-meter walks, with no interaction observed between these 2 factors. Fitbit One was the only monitor with high criterion validity. This interpretation is based on the Fitbit One’s small mean step count percent error (less than −5.0% or +5.0%), lack of systematic bias, and small limits of agreement, and it was deemed equivalent to a zero step count percent error (equivalence bound of −5.0% to +5.0%). Three of the other monitors (Misfit Shine, New-Lifestyles NL-1000, and Jawbone UP2) exhibited moderate correspondence to the criterion, whereas both the Garmin vívofit 2 and Fitbit Charge had poor correspondence with the criterion. Our results for criterion validity are consistent with previous research by Floegel et al [33] who found that the Fitbit One had the lowest mean step count percent error and outperformed other monitors (StepWatch, Omron HJ-112, Fitbit Flex, and Jawbone UP) when compared with direct observation during a 100-meter walk involving both older adults with and without mobility impairments.

For all activity monitors that we tested, walking with interruptions resulted in greater mean step count percent errors than walking continuously. In addition, the mean step count percent error for interrupted walking was not equivalent to zero (equivalence bound of −5.0% to +5.0%), whereas it was equivalent to zero for continuous walking. We observed a systematic bias in step count errors for both walking conditions; specifically, step count errors increased in proportion to the number of steps taken, and the limits of agreement were wide. Previous studies have not tested interrupted walking in older adults in controlled settings, as we did. However, in previous studies that investigated activity monitors during free-living walking conditions in older adults [29-31,47,48], 5 of 8 consumer-grade hip- and wrist-worn activity monitors were found to overcount steps relative to criterion measures [29-31,47,48]. These results are inconsistent with our finding that consumer-grade activity monitors undercounted steps during continuous and interrupted walking. A possible reason for this discrepancy is that during free-living conditions, movements other than stepping (eg, movements during eating or conversation) may reach accelerations that exceed the monitor algorithm thresholds, causing steps to be erroneously recorded [49]. In support of this notion, Tudor-Locke et al [50] compared the hip- and wrist-worn ActiGraph accelerometers during controlled treadmill walking and in free-living conditions. During treadmill testing, they found that the wrist-worn monitor detected fewer steps than the hip-worn monitor; however, during free-living conditions the wrist-worn monitor counted more steps than the hip-worn monitor.

Regarding self-reported mobility, we found that test-retest reliability and criterion validity of step counting were unaffected by the presence of a self-reported mobility limitation, suggesting that older adults with a self-reported mobility limitation can expect similar performance from the activity monitors tested in this study as older adults with self-reported intact mobility. Consistent with our results, Floegel et al [33] reported that mean step count errors for most monitors they tested were similar and small for older adults with or without walking impairment who did not walk with a cane or walker (StepWatch −4.42% vs −3.45%, Fitbit One −2.59% vs −1.71%, Omron −4.48% vs −3.15%, Fitbit Flex −26.94% vs −16.31%, and Jawbone UP −2.86% vs −8.43%). In contrast to our results, Lauritzen et al [8] reported that mobility limitations decreased activity monitor validity when comparing a small group of walker-dependent older adults in nursing homes to healthy older adults [8]. In that study, lower gait assessment scores were significantly correlated with larger absolute percent errors, whereas longer walk times and larger step counts were significantly correlated with larger absolute percent errors. Our study population differed because participants did not use walking aids.

Previous literature indicates that slow gait speed significantly affects the criterion validity of activity monitors [32]. Simpson et al [32] reported that the Fitbit One, when worn on the hip, recorded zero steps when participants walked at speeds between 0.3 m/s and 0.5 m/s, and it had a mean percent error smaller than 10% only when walking speed was 0.8 m/s and 0.9 m/s [32]. Our participants walked, on average, at 1.2 m/s (intact mobility 1.3 m/s and limited mobility 1.1 m/s); thus, speed should not have negatively impacted activity monitor performance in our study. If participants with a self-reported mobility limitation had very slow gait speed or more severe asymmetries in their gait, we may have detected differences in test-retest reliability and criterion validity of step counts from the monitors based on the self-reported mobility status. Future studies of older adults with slower gait speeds (eg, 0.4 m/s to 0.8 m/s) are still needed to understand the performance of consumer-grade activity monitors in the growing population of older adults living with frailty and more severe mobility limitation than this study population.

### Limitations and Strengths

This study had certain limitations. First, the results have limited generalizability with respect to the activity monitors. We tested a single monitor of each activity monitor model with a relatively small sample size. Thus, the results obtained from this study may not be applicable to all versions of the activity monitor model tested or other monitors produced by the same brand. A poorly calibrated monitor (in relation to the average monitor) would have led us to underestimate monitor validity, whereas a better-than-average calibrated monitor would have led us to overestimate monitor validity. Ideally, multiple versions of each monitor would have been tested, and the difference between the monitors was assessed. We had to limit the number and distance of walks performed with our older adult study population to manage participant burden and prevent fatigue, so it was not feasible to conduct additional testing. However, we believe that interdevice variation would likely have been minimal based on a systematic review of consumer-grade activity monitors that reported high interdevice reliability for step counts from 4 studies testing 3 Fitbit models (Classic, One, and Ultra; intraclass correlation coefficients ranged from 0.76 to 1.00) [26]. Second, as the reliability of consumer-grade activity monitors had not been previously evaluated in older adults, we chose to begin by assessing test-retest reliability on short, 100-step CWs. Future studies are needed to examine the effects of walking interruptions and longer distances on test-retest reliability. Our results suggest that reliability under these conditions would likely be better for a hip-worn Fitbit monitor, such as the Fitbit One, than for other monitors. Third, we did not consider the contributions of sex, walking speed, participant height, or stride length on test-retest reliability or criterion validity. In addition, we did not investigate how common daily tasks, other than walking, might affect activity monitor step counts. It will be important for future studies to evaluate the reliability and validity of step counting by consumer-grade activity monitors during a wider range of daily movements than was tested in this study. Further, future studies should seek to determine sources of error during activities of daily living, which often result in overcounting during free-living assessment of consumer-grade activity monitors.

This study also has several strengths. First, all walking tests were performed during overground walking, which represented natural walking conditions more closely than treadmill walking. Treadmill walking has been used frequently in previous studies to evaluate the measurement properties of activity monitors because it enables monitors to be tested at controlled walking speeds. However, older adults who are unfamiliar with treadmill walking exhibit increased heart rate and oxygen consumption while walking on a treadmill compared with overground walking [51]. Moreover, treadmills impose greater symmetry in gait than may be observed naturally, which could, in turn, influence the measurement of reliability and validity. Second, this study tested 6 different activity monitors, and, to our knowledge, 4 of the 6 (Fitbit Charge, Garmin vívofit 2, Jawbone UP2, and New-Lifestyles NL-1000) have not been previously tested in older adults. Unfortunately, because of fast product cycles for consumer-grade monitors, only the Misfit Shine and the New-Lifestyles NL-1000 are currently available for purchase; Fitbit advanced from the One to Inspire and from the Charge to Charge 4, Garmin replaced the vívofit 2 with vívofit 4, and Jawbone went out of business. Nevertheless, the strength of this study is that it presents systematic methods that other researchers can adopt or modify to evaluate the performance of current versions of consumer-grade activity monitors before their use in trials, observational studies, or surveillance systems. Finally, we studied older adults with self-reported mobility limitations, which is important because they are a relevant population for physical activity interventions and surveillance and comprise a sizable proportion of the older adult population.

### Conclusions

The results of this study contribute to the growing literature on consumer-grade activity monitors in the older adult population. This study provides information about the test-retest reliability and criterion validity of step counting by several consumer-grade activity monitors in older adults with either self-reported intact or limited mobility. The results of this study may assist in the selection of an activity monitor for future studies designed to detect changes in physical activity levels, assess adherence to physical activity programs, quantify daily physical activity patterns (in conjunction with self-report questionnaires), or motivate physical activity behavior via goal setting in older adult study populations.

We found variations in step count test-retest reliability and criterion validity across 6 consumer-grade activity monitors when worn by a sample of older adults with self-reported intact and limited mobility. Walk interruptions increased the step count error for all monitors but did not affect any monitor to a greater extent than the others. The presence of self-reported mobility limitations did not affect the step count error. Only one monitor exhibited both high test-retest reliability and criterion validity, the hip-worn Fitbit One, and it is recommended for use in groups of older adults with self-reported intact and limited mobility.

## Abbreviations

ANOVA: analysis of variance
CW: continuous walk
HSD: honest significant difference
IW: interrupted walk
RW: reliability walk
SEM: standard error of measurement
SPPB: Short Physical Performance Battery
